# Understanding the Correlation between Tomographic and Biomechanical Severity of Keratoconic Corneas

**DOI:** 10.1155/2015/294197

**Published:** 2015-04-06

**Authors:** Rohit Shetty, Rudy M. M. A. Nuijts, Purnima Srivatsa, Chaitra Jayadev, Natasha Pahuja, Mukunda C. Akkali, Abhijit Sinha Roy

**Affiliations:** ^1^Cornea and Refractive Surgery, Narayana Nethralaya, Bangalore 560010, India; ^2^Cornea Clinic, Department of Ophthalmology, Maastricht University Medical Center, 6211 LK Maastricht, The Netherlands; ^3^Imaging, Biomechanics and Mathematical Modeling Solutions, Narayana Nethralaya, Bangalore 560099, India

## Abstract

*Purpose.* To evaluate correlation between tomographic gradation of keratoconus (KC) and its corresponding air-puff induced biomechanical response. * Methods.* Corneal tomography and biomechanics were measured with Scheimpflug imaging in 44 normal and 92 KC corneas. Deformation waveform was also analyzed with Fourier series. A custom KC severity scale was used from 1 to 3 with 3 as the most severe grade. Tomographic and biomechanical variables were assessed among the grades. Sensitivity and specificity of the variables were assessed using receiver operating characteristics (ROC). * Results.* Curvature variables were significantly different between normal and disease (*P* < 0.05) and among grades (*P* < 0.05). Biomechanical variables were significantly different between normal and disease (*P*<0.05) but similar among grades 1 and 2 (*P* > 0.05). All variables had an area under the ROC curve greater than 0.5. The root mean square of the Fourier cosine coefficients had the best ROC (0.92, cut-off: 0.027, sensitivity: 83%, specificity: 88.6%). Spearman correlation coefficient was significant between most variables (*P* < 0.05). However, tomographic segregation of keratoconus did not result in concomitant biomechanical segregation of the grades. * Conclusions.* There was lack of significant biomechanical difference between mild disease grades, despite progressive corneal thinning. Mathematical models that estimate corneal modulus from air-puff deformation may be more useful.

## 1. Introduction

The hypothesis that the biomechanical strength of the cornea needs to be restored forms the basis of various treatment modalities used in the management of keratoconus [1]. Corneal transplantation using normal corneal tissue was one of the obvious options to restore vision in affected patients. In recent times, ultraviolet (UV-A) collagen crosslinking with a photosensitive crosslinking agent was used widely to restore the biomechanical strength of the cornea [1, 2]. This treatment resulted in flattening of the zone of focal biomechanical weakening [1] and a concomitant reduction in corneal wavefront aberrations [3, 4]. To treat keratoconus, an alternate procedure combined topography guided photorefractive keratectomy and UV-A crosslinking [5]. The combined treatment resulted in better visual outcomes than crosslinking alone though no information was available on the postoperative biomechanical status of the keratoconic cornea [6].

Prior to selecting the appropriate treatment, it was important to evaluate the preoperative biomechanical status of the cornea [2]. There was evidence that crosslinking alone provided better outcomes in early and mild keratoconus but not necessarily in advanced cases [7]. Additionally, most studies used a definition of “progression” that was based on documented topographic steepening over a period of six months to a year [5]. This progression of the disease may also be considered to be an indicator of progressive biomechanical weakening. However, this hypothesis remained untested in KC patients. Recently, a high-speed Scheimpflug imaging device, the Corvis-ST (Oculus Optikgerate Gmbh, Germany), was used to detect corneal deformation in response to an air-puff incident on the anterior corneal surface [7]. Corneal deformation (or displacement) was a quantifier of the biomechanical stiffness of the cornea [7] and it was intuitive that biomechanically weaker corneas would deform more. In this study, the biomechanical status of different grades of keratoconus, segregated based on a custom severity scale, was evaluated using the Corvis-ST. The custom severity scale was designed based on the anterior surface mean keratometry value such that it stratified the severity of the KC disease as a linear function of the grade. Further, the study evaluated the relative impact of thinning and decrease in stiffness of the cornea on the progression of the disease.

## 2. Methods

The study was a retrospective, observational study in a tertiary eye care center in southern India. The study protocol was approved by the institutional review board of the center and followed the tenets of the Declaration of Helsinki. The study included 44 (44 subjects) normal and 92 (92 subjects) keratoconic eyes. The diagnosis of keratoconus was based on evidence of stromal thinning on slit-lamp, focal protrusion or increase in corneal curvature, Fleischer's ring, Vogt's striae, scissoring of the red reflex, an abnormal retinoscopy, and curvature asymmetry leading to abnormal corneal astigmatism. Further, the classification of the severity of the keratoconus was performed using corneal tomography. Based on the anterior surface mean keratometry value (Kmean), three keratoconus grades and a normal grade classified as grade 0 were established: grade 1—Kmean < 48 D; grade 2—48 D ≤ Kmean < 52 D; grade 3—Kmean ≥ 52 D [8]. The number of subjects in grades 1, 2, and 3 was 36, 29, and 27 eyes, respectively. The exclusion criteria were glaucoma, a history of eye surgery, or current topical medication use. For “normal” eyes, manifest spherical error and astigmatism were limited to ±2 D.

Corneal tomography was evaluated with the Pentacam (Oculus Optikgerate Gmbh, Germany). The tomography variables that were selected for analyses were steep (K2) and flat (K1) axis keratometry, mean keratometry (Kmean), maximum axial curvature (Kmax), central corneal thickness (CCT), the thickness of the thinnest point of the cornea (TPT), and the location of the cone. The location of the cone was assessed as the distance between the location of peak tangential curvature and the geometric center of tangential curvature map. Kmean was the average of K1 and K2. Biomechanics of the cornea was evaluated with the Corvis-ST (Oculus Optikgerate Gmbh, Germany). The Corvis-ST also had an ultra-high-speed Scheimpflug imaging system that captured 140 frames of a cross-section (along the horizontal meridian) of the deforming cornea over a time period of 30 milliseconds. Advanced edge detection algorithm was used to measure the displacement of the anterior and posterior edge of the deforming cornea. The device reported the displacement of the anterior corneal apex as a function of the application time of air-puff. There were several variables reported by the device based on the measured displacement of the cornea apex. In this study, the following variables were used for analyses of the biomechanical status of corneas: A1—time of first applanation, A2—time of second applanation, Time—time of peak displacement of the corneal apex, DA—deformation amplitude (or magnitude of peak displacement of the corneal apex), IOP—intraocular pressure measured by Corvis-ST.

Further, a Fourier series fit to the displacement of the corneal apex was performed [9]. The Fourier series fit was simply a nonlinear regression of DA versus time [9]. Three variables were defined based on the Fourier coefficients of the regression: AUDA—area under the deformation amplitude curve, “an” RMS—root mean square of cosine Fourier coefficients, “bn” RMS—root mean square of Fourier sine coefficients [9]. Fourier coefficients up to order 31 were used for the Fourier series fit. With AUDA being a measure of biomechanical status of the cornea, a larger AUDA implied a biomechanically weaker cornea and vice versa [9]. Similarly, an RMS and bn RMS were expected to be greater in weak corneas compared to normal corneas [9].

### 2.1. Statistical Analyses

The variables were tested for normality of distribution. Since variables were observed to be nonparametrically distributed, all continuous variables were reported as median ±95% confidence interval (CI). Difference between the grades was assessed with Kruskal-Wallis test followed by post hoc analyses. Correlation between all the variables was assessed with the Spearman correlation coefficient. The sensitivity and specificity of each variable to detect keratoconus were analyzed with receiver operating characteristic (ROC). A *P* value less than 0.05 was considered to be statistically significant. MedCalc v12.5.0 (MedCalc Inc., Belgium) was used for statistical analyses.

## 3. Results

**Figure 1:**
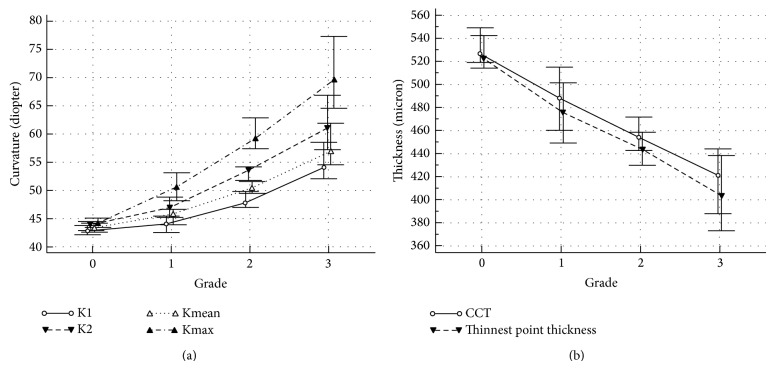
(a) Median curvature in diopters (simulated keratometry (K1, K2), mean curvature (Kmean), and maximum curvature (Kmax) as a function of KC grade. Grade 0 implies unaffected eyes; (b) median central corneal thickness (CCT) and thickness of thinnest point as a function of grade.

Table 1 lists the median and 95% CI for all the variables. All variables increased in magnitude with increasing severity of keratoconus (Table 1). Figures 1(a) and 1(b) show the median curvature and thickness of the different grades. The severity scale graded the curvature and thickness of keratoconic corneas as a linear function of grade number (Figures 1(a) and 1(b)). Statistical analyses of curvature and thickness yielded a statistically significant difference between the grades. Kmax of grades 0, 1, 2, and 3 were significantly different from each other (*P* < 0.0001). Also, Kmean, K1, and K2 differed significantly among the grades (*P* < 0.0001). Both CCT and TPT differed significantly among the grades as well (*P* < 0.0001). The location of the cone was similar among all keratoconus grades (*P* = 0.25).

**Figure 2:**
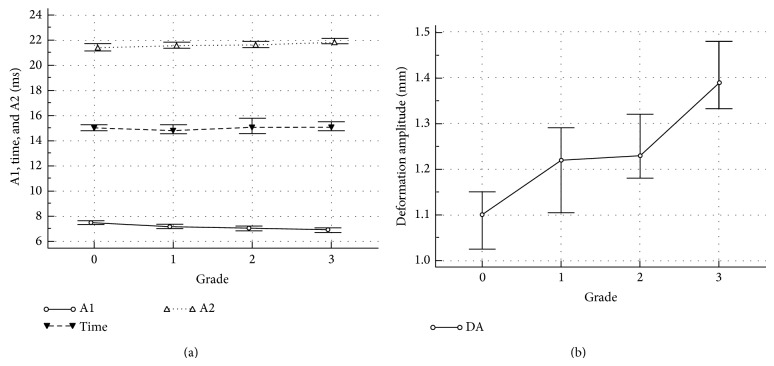
(a) Time of first applanation (A1), peak applanation (Time), and second applanation (A2) as a function of KC grade. Grade 0 implies unaffected eyes. All units are msec; (b) deformation amplitude in mm as a function of KC grade. All values are the medians ±95 CI.

**Figure 3:**
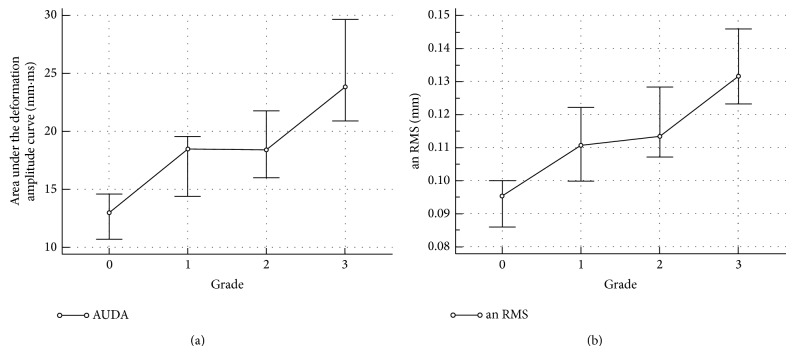
(a) Area under the deformation amplitude curve in mm·mesc as a function of KC grade. Grade 0 implies unaffected eyes; (b) an RMS, the root mean square of Fourier cosine coefficients, as a function of grade. All values are the medians ±95 CI.

The biomechanical parameters were also evaluated. Figures 2(a) and 2(b) show the median values of A1, Time, A2, and deformation amplitude (DA) of the grades. While A1 decreased, A2 and Time increased with increasing severity of keratoconus (Table 1). A1 of grade 0 was significantly different from other grades (*P* < 0.0001). A1 of grade 1 was similar to grade 2 (*P* > 0.05) but not to grade 3 (*P* < 0.05). Time did not differ significantly among the grades (*P* = 0.83). A2 of grades 0 and 1 were similar (*P* > 0.05). A2 of grades 2 and 3 were similar (*P* > 0.05) but were different from grades 0 and 1 (*P* < 0.05). DA also differed among the groups. DA of grade 0 differed from all other grades (*P* < 0.001). While DA of grades 1 and 2 were similar (*P* > 0.05), DA of grade 4 differed significantly from other grades (*P* < 0.05). Figures 3(a) and 3(b) show the median values ±95% CI of AUDA and an RMS of all grades. AUDA of grades 0 and 4 differed significantly from other grades and from each other (*P* < 0.05). However, AUDA of grades 1 and 2 were similar to each other (*P* > 0.05) and differed from grades 0 and 4 (*P* < 0.05). Similar trends were observed with an RMS but bn RMS of all grades were similar to each other (*P* > 0.05). IOP of grade 0 was significantly greater than all keratoconus grades (*P* < 0.001). However, IOP of grades 1, 2, and 3 were similar (*P* > 0.05).

**Figure 4:**
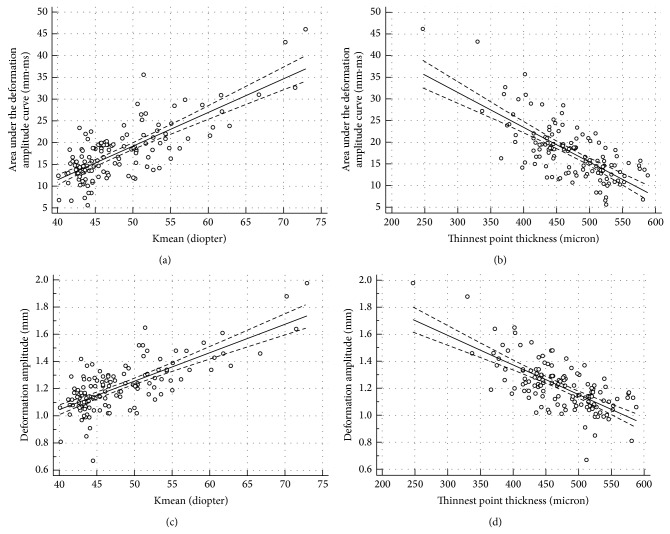
(a) Area under the deformation amplitude curve (AUDA) versus mean keratometry (Kmean); (b) area under the deformation amplitude curve (AUDA) versus thinnest point thickness (TPT); (c) deformation amplitude curve (DA) versus mean keratometry (Kmean); (d) deformation amplitude curve (DA) versus thinnest point thickness (TPT). The solid line is the linear regression of the data. The dotted lines are 95% CI limits of the regressed line.

**Figure 5:**
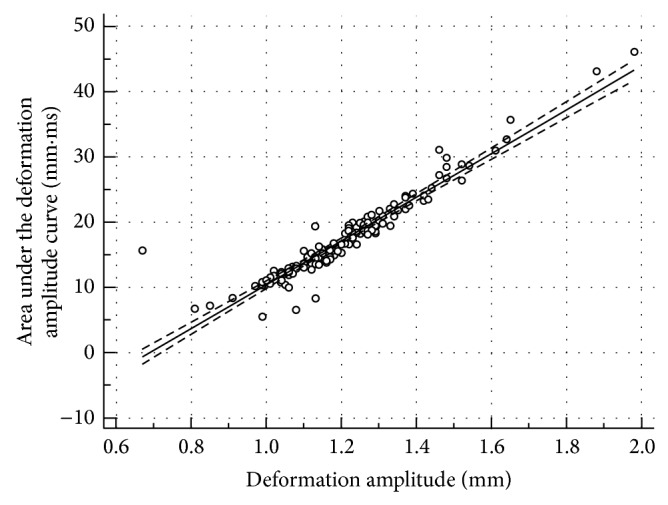
Area under the deformation amplitude curve (AUDA) versus deformation amplitude (DA).

**Figure 6:**
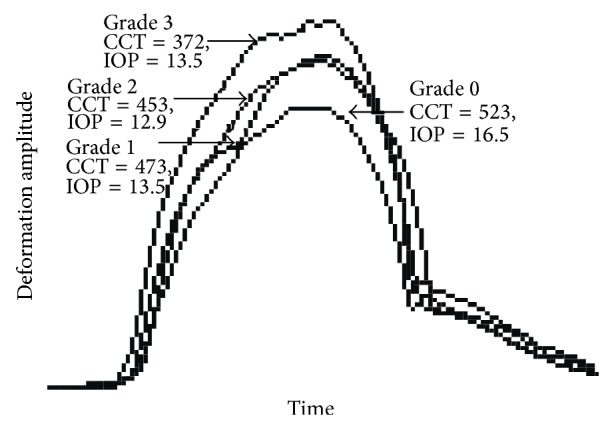
Overlay of deformation amplitude of four corneas, one from each grade. The corneal thickness (CCT) and intraocular pressure (IOP) are reported next to the grade label.

The correlation between all variables was assessed with the Spearman correlation coefficient (Table 2). Most of the correlations were statistically significant (*P* < 0.05). Keratometry correlated well with all biomechanical variables (*P* < 0.001). Interestingly, AUDA and an RMS had a very high correlation (0.945 and 0.919) with DA. As an example, Figures 4(a), 4(b), 4(c), and 4(d) show the linear regression of AUDA and DA with Kmean and TPT. Both AUDA and DA had a significantly negative correlation with Kmean and TPT.  Figure 5 shows the correlation between AUDA and DA using all the grades.  Table 3 lists the results from the ROC analyses. Time had the least area under the ROC curve equal to 0.511 with a sensitivity and specificity of 70.5% and 62.8%, respectively. Since keratometry was used for gradation, it had the highest area under the ROC curves among all variables (greater than 0.9). Among the biomechanical variables, an RMS had the best area under the ROC curve equal to 0.915 with sensitivity and specificity of 83% and 88.6%, respectively. AUDA was a close second (area under the ROC curve = 0.886, sensitivity = 73.9%, specificity = 93.2%).  Figure 6 shows an overlay of DA of four corneas, one from each grade, with CCT and IOP reported next to the grade label. From Figure 6, salient observations relative to grade 0 were as follows: (a) quicker increase in DA in the first half of the applanation test in higher disease grades; (b) DA was greater in higher disease grades; (c) slower decrease in DA in the second half of the applanation test in higher disease grades; (d) globe deformation was similar in all the corneas.

## 4. Discussion

Corneal tomography attracted a lot of attention as the primary diagnostic tool for keratoconus [10–14]. Steepening of the cornea coupled with thinning of the stroma and epithelium [15] contributed to the worsening vision in keratoconus patients. The steepening of the cornea was an end result of both biomechanical weakening and thinning of the cornea [1]. Several new tomographic indices were postulated for improved detection of keratoconus, with higher sensitivity and specificity [10–14]. A recent study in a large cohort of keratoconus subjects demonstrated that anterior surface irregularity indices were better in diagnosing the disease in early stages than visual acuity and pachymetry [16]. Thus further studies are needed to assess the sensitivity and specificity of these new tomographic variables in other centers. To assess the biomechanical weakening of the cornea, the ocular response analyzer (ORA) was the first device available commercially. It was extensively used to study the biomechanical status of keratoconus [17–22]. The ORA did not report corneal deformation. However, the reflected beam intensity from the anterior surface of the cornea was considered to be representative of corneal deformation. Based on the ORA waveform, corneal hysteresis (CH) and corneal resistance factor (CRF) were lower in KC eyes, with a sensitivity and specificity below 80% [17], and insensitive to corneal stiffening caused by collagen crosslinking [18]. Thus, the true relationship of CH and CRF to the biomechanical status of the cornea, such as Young's modulus, was not understood.

The grading system used in this study was based on the data reported in a past study on keratoconus using the ORA [8]. In that study, CH and CRF did not produce statistically significant biomechanical difference between the grades [8]. Even though CH and CRF were insensitive to collagen crosslinking, each ORA waveform had characteristic features that may reveal the biomechanical status of keratoconic corneas [19–22]. These studies introduced new variables that had better sensitivity and specificity compared to CH and CRF in the detection of keratoconus [19–22], like the hysteresis loop area [22], which was similar by definition to AUDA. Some of the common conclusions from ORA studies on keratoconus included an earlier applanation, lower pressure required to cause applanation, lower signal peak, and delayed recovery of the ORA signal after minimum concavity was attained in keratoconic corneas. However similar to CH and CRF, these new variables did not report any significant biomechanical differences between grades of keratoconus [19–22]. Thus, the physical meaning of these new ORA variables was also undetermined.

Similar to the ORA, the Corvis-ST showed earlier applanation (A1 decreased), greater deformation (DA increased), delayed recovery of the cornea (A2 increased), and lower biomechanical response (DA and AUDA increased) with increasing disease severity (Figure 6). The root mean square variables, which were a measure of the undulations or noise in the signal [9], also increased with increasing disease severity. The correlation between the variables was also significant (Table 2). Interestingly, the correlation between AUDA and DA was very high, indicating that if DA was known a priori, AUDA may be estimated with more than 90% accuracy in keratoconus. The sensitivity and specificity of the biomechanical variables were in excess of 80% but lower than the topographic variables in this study and those reported in other studies [10–14]. In this study, IOP was similar among all keratoconus grades but was significantly different from normal corneas. In biomechanically normal corneas, decrease in IOP from 36 to 15 mmHg resulted in a keratometric decrease of 1 D [23]. In keratoconic corneas, increase in IOP from 16 to 36 mmHg resulted in a keratometric increase of 4.1 D [24]. By linear interpolation, this may imply that keratometry would change by 0.8-0.9 D in keratoconus eyes for a 4-5 mmHg change in IOP in the physiological range. However, IOP was similar among all disease grades in this study (*P* > 0.05). Since IOP was not responsible for increase in keratometry between the grades (1, 2, and 3), it may be concluded that tomographic and biomechanical changes were responsible for disease progression. Since the area under the curve from the ROC analyses of the biomechanical variables was lower than tomography, the biomechanical variables in this study may not be sensitive to early changes in the biomechanical status of the KC cornea compared to tomography [16].

Since deformation amplitude of grades 0 and 3 was significantly different from grades 1 and 2, it may be concluded that corneal deformation reported by Corvis-ST may be more representative of the biomechanical state of keratoconic corneas than the ORA variables. Further, DA, AUDA, and an RMS demonstrated similar biomechanical response of grades 1 and 2, which may explain why crosslinking halts or delays the progression of disease in early and mild keratoconic corneas but not in advanced cases (grade 3) [7]. In advanced cases, the biomechanical stiffness may be too low to be compensated by the magnitude of stiffening caused by crosslinking. While tomography worsened nearly linearly from grade 1 to grade 3, grades 1 and 2 had similar AUDA, DA, and an RMS but the same differed from grade 3. Thus, thinning of the cornea may be one of the drivers of disease progression from grade 1 to grade 3, while the viscoelastic properties of the cornea may have remained similar across grades 1 and 2. Another study on keratoconic corneas using the Corvis-ST also reported greater DA in keratoconus but the sensitivity of DA to detect the disease was 0.77 [25].

Deformation of the corneal apex is a sum of both the corneal and globe deformation. The globe deformation was only about 1/10th of the measured deformation amplitude at its peak value [26]. Therefore, globe deformation was unlikely to influence the outcomes of this study using the investigated variables. Refined mathematical models based on continuum soft tissue mechanics may be required such that a measure of Young's modulus or nonlinear modulus could be defined [26–28]. The device has a limited depth resolution and finer biomechanical abnormalities in the corneal stroma cannot be measured. Thus, anisotropy of the cornea may not be measured accurately, for example, depth variation in mechanical strain due to crosslinks between collagen lamellas [29]. Techniques to resolve the depth dependent differences in the biomechanical strength of the cornea are in development [30–32]. The air-puff caused deformation of the cornea up to a radius of 3 mm from its geometric center [26, 27]. Hence keratoconic cones beyond the 3 mm radius central cornea may not undergo any deformation. A recent study showed that both ORA and Corvis-ST may be required to differentiate between pellucid marginal degeneration (PMD) and normal corneas [33]. However, the devices were unable to distinguish between keratoconus and PMD [33]. In practice, both devices assessed the central cornea and the outcomes of the recent study [33] were confusing. This highlighted the need of advanced analysis methods [26–28] or measurement tool [30–32] that may perform cone location specific measurements. In this study, cone location was unlikely to influence the study outcomes as there was no significant difference (*P* > 0.05) in cone location among the grades of keratoconus. In conclusion, corneal deformation by Corvis-ST was a direct measure of the biomechanical status of the cornea and may aid to accurately quantify the grades of keratoconus. Separate biomechanical grading scale of keratoconus severity is the need of the hour in addition to traditional tomographic grading.

## Figures and Tables

**Table 1:** Median ± 95% CI of the variables evaluated in normal corneas and diseases grades.

	Normal		KC					
	Grade 0		Grade 1		Grade 2		Grade 3	
	Median	95% CI	Median	95% CI	Median	95% CI	Median	95% CI
*K*1 (D)	42.7	42.4–43.2	44	43.3–44.5	47.7	47.2–49.0	54	52.2–58.0
*K*2 (D)	43.7	43.2–44.2	46.8	45.8–47.8	53.4	52.4–53.8	60.9	57.5–65.5
*K*max (D)	44.2	43.6–44.6	50.6	48.9–52.5	59.2	58.4–60.6	69.6	65.4–75.2
*K*mean (D)	43.3	42.7–43.6	45.8	44.5–46.1	50.4	50.0–51.3	56.9	54.9–61.3
Location of cone (mm)	—	—	0.28	0.12–1.53	0.25	0.16–1.48	0.25	0.12–1.15
CCT (micron)	526.5	521.0–536.9	488	466.6–505.0	454	446.7–464.7	421	391.4–440.3
TPT (micron)	522	517.0–528.9	475	459.3–494.7	443	435.5–448.1	403	376.0–432.6
IOP (mmHg)	16.3	15.5–17.0	14	13.5–14.7	13.5	12.0–14.0	12.5	11.2–13.0
*A*1 (msec)	7.48	7.41–7.55	7.15	7.08–7.26	7.06	6.88–7.15	6.92	6.74–7.03
Time (msec)	15.02	14.78–15.25	14.78	14.78–15.10	15.02	14.73–15.53	15.02	14.78–15.40
*A*2 (msec)	21.41	21.26–21.57	21.56	21.42–21.74	21.63	21.51–21.81	21.85	21.73–22.05
DA (mm)	1.10	1.06–1.13	1.22	1.15–1.26	1.23	1.21–1.30	1.39	1.34–1.47
AUDA (mmHg·msec)	13.01	12.09–13.84	18.5	15.72–19.41	18.42	16.70–20.99	23.84	21.13–28.64
an RMS (mm)	0.095	0.091–0.099	0.111	0.107–0.118	0.113	0.109–0.124	0.132	0.125–0.142
bn RMS (mm)	0.034	0.033–0.037	0.026	0.023–0.029	0.028	0.023–0.032	0.030	0.025–0.039

**Table 2:** Spearman correlation coefficients to compare correlation between the variables. The *P* values are listed for each correlation and represent the statistical significance of the correlation.

	*A*1	Tune	*A*2	DA	IOP	AUDA	an RMS	bn RMS	TPT	CCT	*K*1	*K*2	*K*mean	*K*max
*A*1		−0.244	−0.741	−0.793	0.959	−0.843	−0.818	0.104	0.56	0.566	−0.475	−0.568	−0.605	−0.547
		0.0048	<0.0001	<0.0001	<0.0001	<0.0001	<0.0001	0.2367	<0.0001	<0.0001	<0.0001	<0.0001	<0.0001	<0.0001

Time			0.289	0.198	−0.25	0.217	0.169	−0.31	0.04	0.029	0.004	0.06	0.038	0.03
			0.0008	0.0228	0.0038	0.0125	0.0531	0.0003	0.6465	0.7394	0.967	0.4943	0.6669	0.7352

*A*2				0.756	−0.72	0.754	0.745	0.078	−0.335	−0.364	0.345	0.388	0.372	0.376
				<0.0001	<0.0001	<0.0001	<0.0001	0.3728	0.0001	<0.0001	0.0001	<0.0001	<0.0001	<0.0001

DA					−0.753	0.957	0.954	0.083	−0.648	−0.659	0.541	0.662	0.671	0.63
					<0.0001	<0.0001	<0.0001	0.3464	<0.0001	<0.0001	<0.0001	<0.0001	<0.0001	<0.0001

IOP						−0.806	−0.785	0.098	0.529	0.535	−0.452	−0.545	−0.576	−0.523
						<0.0001	<0.0001	0.2638	<0.0001	<0.0001	<0.0001	<0.0001	<0.0001	<0.0001

AUDA							0.949	0.027	−0.66	−0.667	0.577	0.685	0.693	0.659
							<0.0001	0.7596	<0.0001	<0.0001	<0.0001	<0.0001	<0.0001	<0.0001

an RMS								0.04	−0.702	−0.718	0.581	0.705	0.716	0.67
								0.6516	<0.0001	<0.0001	<0.0001	<0.0001	<0.0001	<0.0001

bn RMS									0.047	0.012	−0.065	−0.139	−0.165	−0.119
									0.5932	0.8942	0.4565	0.1123	0.0593	0.1757

TPT										0.976	−0.714	−0.804	−0.793	−0.785
										<0.0001	<0.0001	<0.0001	<0.0001	<0.0001

CCT											−0.726	−0.805	−0.788	−0.79
											<0.0001	<0.0001	<0.0001	<0.0001

*K*1												0.876	0.85	0.955
												<0.0001	<0.0001	<0.0001

*K*2													0.939	0.974
													<0.0001	<0.0001

*K*mean														0.929
														<0.0001

*K*max														
													

**Table 3:** Receiver operator characteristics (ROC) curve for each variable. The area under the ROC curve with 95% CI in brackets, cut-off, sensitivity, and specificity are listed for each column.

	Area under the ROC curve	Cut-off	Sensitivity	Specificity
*A*1	0.87 (95% CI: 0.80, 0.92)	<21.48	68.2	88.6
Time	0.51 (95% CI: 0.42, 0.60)	>15.71	70.5	62.8
*A*2	0.69 (95% CI: 0.42, 0.60)	>1.17	19.3	97.7
DA	0.86 (95% CI: 0.79, 0.91)	>16.59	76.1	86.4
AUDA	0.89 (95% CI: 0.82, 0.94)	>0.104	73.9	93.2
an RMS	0.92 (95% CI: 0.85, 0.96)	<0.027	83.0	88.6
bn RMS	0.69 (95% CI: 0.61, 0.77)	<0.027	53.4	88.6
*K*1	0.88 (95% CI: 0.80, 0.92)	>44.3	77.3	93.2
*K*2	0.95 (95% CI: 0.89, 0.98)	>45.3	88.2	95.5
*K*mean	0.93 (95% CI: 0.88, 0.97)	>45.4	80.7	100.0
*K*max	0.99 (95% CI: 0.97, 1.0)	>46.5	95.5	100.0
CCT	0.93 (95% CI: 0.87, 0.96)	<516	92.0	84.1
TPT	0.86 (95% CI: 0.84, 0.97)	<509	83.3	84.1
